# The Role of Leaky Gut in Functional Dyspepsia

**DOI:** 10.3389/fnins.2022.851012

**Published:** 2022-03-29

**Authors:** Lucas Wauters, Matthias Ceulemans, Jolien Schol, Ricard Farré, Jan Tack, Tim Vanuytsel

**Affiliations:** ^1^Department of Gastroenterology and Hepatology, University Hospitals Leuven, Leuven, Belgium; ^2^Translational Research Center for Gastrointestinal Disorders (TARGID), Department of Chronic Diseases and Metabolism, KU Leuven, Leuven, Belgium

**Keywords:** functional dyspepsia, permeability, immunology, duodenum, gut-brain-axis

## Abstract

Patients with functional dyspepsia (FD) complain of epigastric symptoms with no identifiable cause. Increased intestinal permeability has been described in these patients, especially in the proximal small bowel or duodenum, and was associated with mucosal immune activation and symptoms. In this review, we discuss duodenal barrier function, including techniques currently applied in FD research. We summarize the available data on duodenal permeability in FD and factors associated to increased permeability, including mucosal eosinophils, mast cells, luminal and systemic factors. While the increased influx of antigens into the duodenal mucosa could result in local immune activation, clinical evidence for a causal role of permeability is lacking in the absence of specific barrier-protective treatments. As both existing and novel treatments, including proton pump inhibitors (PPI) and pre- or probiotics may impact duodenal barrier function, it is important to recognize and study these alterations to improve the knowledge and management of FD.

## Introduction

Functional dyspepsia (FD) is a common gastrointestinal (GI) disorder with unknown pathophysiology. According to the Rome IV criteria, two subgroups of FD were proposed: postprandial distress syndrome (PDS) with postprandial fullness or early satiation, and epigastric pain syndrome (EPS) with epigastric pain or burning (Stanghellini et al., 2016). Symptoms must be severe enough to impact on usual activities with a minimal frequency of 1 (EPS) or 3 (PDS) days per week, and be present for the past 3 months with symptom onset at least 6 months before diagnosis (Stanghellini et al., 2016). In contrast, organic dyspepsia may result from erosive esophagitis, peptic ulcer disease or cancer in a minority of patients (Ford et al., 2010; Enck et al., 2017; Ford et al., 2020). While 7–10% of adults fulfilled Rome IV criteria for FD in large internet-based cross-sectional health surveys, organic pathology was not systematically ruled out by endoscopy (Aziz et al., 2018; Sperber et al., 2021), which is expected to be normal in approximately 80% of dyspeptic patients (Ford et al., 2010).

Despite the absence of macroscopic alterations during investigation, recent findings point toward microscopic alterations in the duodenal mucosa of FD patients (Wauters et al., 2020b). The duodenum or proximal small intestine has emerged as a key player in disorders of gut-brain interaction (DGBI) as it regulates the passage of food as chyme from the stomach to the small intestine, where nutrients are absorbed (van Baar et al., 2018). Auto- and paracrine mechanisms in the duodenum are also involved in the mucosal defense to acid and luminal digestion of nutrients with secretion of bile and pancreatic juice (Rønnestad et al., 2014). Besides nutrient sensing, passage of luminal content is taking place in the proximal small intestine, which is the most permeable region with the largest intercellular pores of the GI-tract (Camilleri, 2019). Moreover, the small bowel microenvironment allows for a closer interaction between the lumen and host cells in comparison to the colon (Donaldson et al., 2016).

Increased duodenal permeability and immune cell infiltration have been repeatedly reported in FD patients (Wauters et al., 2020b). While the occurrence of duodenal pathology is increasingly recognized, potential luminal (food, acid, bile and the microbiota) or central (stress) causes are still unclear (Wauters et al., 2020a; Wauters et al., 2021c). Duodenal functionality balances between nutrient absorption through an epithelial monolayer on the one hand, while at the same time forming a highly performant barrier that prevents the leakage of luminal content through the epithelium. This is summarized as the delicate but vital balance between intestinal permeability and barrier function (Vanuytsel et al., 2021). In this review, we discuss the different players and measurements of duodenal barrier function and provide a comprehensive overview of studies performed in FD patients, along with potentially associated or causal factors.

## Duodenal Barrier Function

### Luminal Barrier

**FIGURE 1:**
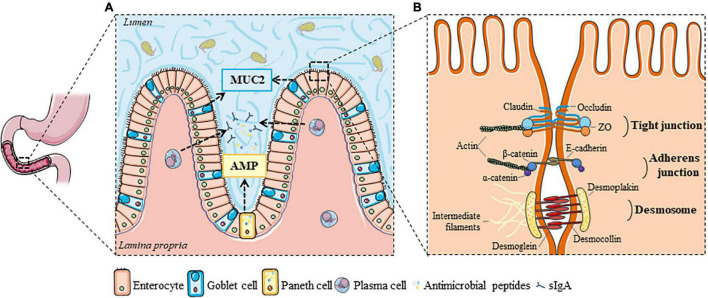
Luminal and cellular elements of the duodenal barrier. **(A)** The intestinal lumen is separated from the epithelium by a mucus layer formed by mucin glycoproteins, mainly MUC2 from goblet cells, in which bacterial residence is limited by the secretion of antimicrobial peptides (from Paneth or epithelial cells) and sIgA (from plasma cells). **(B)** Tight junctions, adherens junctions and desmosomes are important cell-to-cell adhesion proteins that regulate the epithelial barrier function, and initiate and stabilize enterocyte adhesion in the gut. AMP, antimicrobial peptides; MUC2, Mucin 2; sIgA, secretory immunoglobulin A; ZO, zonula occludens. This figure was created with elements from Smartservier.

The duodenal barrier is formed by the complementarity of two main elements: the luminal and the tissue compartment (Farré and Vicario, 2017). The luminal compartment comprises various factors, such as bile, gastric acid and pancreatic juice that make the duodenal lumen a hostile environment for micro-organisms (Camilleri, 2019). The first physical barrier that protects the epithelium from toxins and pathogens is the mucus layer, consisting of a mucin glycoprotein network secreted by goblet cells (Figure 1A). The duodenal mucin network is, in contrast to that of the stomach and colon, a loosely and unattached gel-network that separates the lumen from the epithelium (Johansson et al., 2013). The mucus layer is mainly formed by mucin 2 (MUC2), a large and highly glycosylated protein that is abundant in the GI-tract (Johansson et al., 2013). Besides mucins, a variety of antimicrobial peptides is secreted in the mucus layer mainly by Paneth cells in the duodenal crypts, which limit the bacterial residence in the duodenal mucus (Johansson et al., 2013). Antimicrobial factors include secretory immunoglobulin A (sIgA) released by plasma cells, α-defensins by Paneth cells, and β-defensins by epithelial cells (Figure 1A; Camilleri, 2019; Hooper and MacPherson, 2010).

### Cellular Barrier

The different layers of the duodenal wall constitute the tissue compartment. Enterocytes make up around 80% of the cells of the epithelium (Figure 1A). They function as a physical barrier, regulate nutrient sensing and uptake, and are involved in immunological processes. The enterocyte lining is interspersed with goblet, Paneth, enterochromaffin and microfold or membranous (M-)cells found in the gut-associated lymphoid tissue (GALT) of the Peyer’s patches, which all aid in maintaining the immunological barrier in the duodenum (Camilleri, 2019; Farré and Vicario, 2017). The duodenal wall is highly folded and has numerous villi and crypts to maximize the contact surface with the lumen and increase the absorption of nutrients. Luminal contents can pass the epithelium *via* different transport routes, depending on their physicochemical properties, of which the paracellular route is the most commonly studied (Farré and Vicario, 2017). Three major cell-to-cell adhesion structures were first described by Farquhar and Palade: tight junctions (TJ), adherens junctions (AJ) and desmosomes (Figure 1B; Farquhar and Palade, 1963). Paracellular transport is strictly controlled to prevent leakage of water and solutes and uncontrolled penetration of luminal substances. This is especially performed by TJ complexes, consisting of the transmembrane proteins occludin (OCLN) and claudins (CLDN), which connect to zonula occludens (ZO) complexes and anchor them to the actin cytoskeleton (Farré and Vicario, 2017; Odenwald and Turner, 2017). The composition and the amount of transmembrane proteins in the TJ determine the permeability of the pores (Shen et al., 2011). While OCLN and ZO are involved in the so-called paracellular leak pathway, the CLDN family regulates the pore pathway as discussed below (Shen et al., 2011; Zuo et al., 2020).

Further downward from the apical side, AJ and desmosomes support the formation of TJ by their adhesive forces (Figure 1B). E-cadherins are important integral membrane proteins in epithelial AJ and interact with β-catenin, which in turn is linked to the actin cytoskeleton *via* α-catenin (Odenwald and Turner, 2017). Desmosomes are comprised of desmoglein (DSG) and desmocollin (DSC), two cadherin-type molecules that link intermediate filaments of adjacent cells *via* interaction with desmoplakin and anchor the epithelium by connecting intermediate filaments to the underlying basal lamina (Farré and Vicario, 2017). Together, these three junctional complexes are important regulators of duodenal integrity. Besides the paracellular route, the transcellular endocytic route is another major but separate pathway and both can be measured using different methodologies.

## Measuring Intestinal Permeability

### Functional Assessment: *in vivo*

The most common permeability test is the differential urinary sugar excretion test, which consists of a urine collection after ingestion of sugars which are not metabolized and renally eliminated after intestinal absorption (Camilleri and Vella, 2021). The preferred sugars are mono- (mannitol and rhamnose) and disaccharides (lactulose and sucralose) and the collection period of 0–2 h was validated for the small bowel, as colonic permeability is measured with increasing time (Rao et al., 2011). Besides the lactulose–mannitol ratio (LMR) as a marker of small intestinal permeability, urinary sucrose excretion has been used to measure for gastroduodenal permeability (Table 1; Mujagic et al., 2014). While lactulose and mannitol may be markers for paracellular and transcellular passage, respectively, supporting evidence is lacking (Blomquist et al., 1993). It was also proposed that lactulose passes the intestinal epithelium *via* the paracellular leak and mannitol *via* the pore pathway (see “Functional Assessment: *ex vivo*”), but this concept is controversial (Camilleri, 2019). Large polyethylene glycols (PEG) (>40 kDa) have been used in the past to assess the intestinal permeability in the context of inflammatory bowel diseases (IBD) but these probes are likely to cross the epithelium *via* the transcellular route based on the high molecular weight (Vanuytsel et al., 2021).

**TABLE 1:** Techniques and measurements of duodenal barrier function in functional dyspepsia.

Type	Technique	Measurements
*Functional (in vivo)*	Differential urinary sugar test	LMR (0–2 h)sucrose excretion
	Electrical resistance	Mucosal admittance or impedance
	Confocal laser endomicroscopy	Epithelial gap density (cell extrusion zones)
	Blood markers	LPS-binding protein (translocation), IFABP (damage), zonulin (regulator)
*Functional (ex vivo)*	Ussing chambers	TEER, conductance
		Basal ion transport (Isc)
		Passage or flux:
		- paracellular (4–20 kDa labeled dextrans)
		- transcellular (40–80 kDa, bacteria)
*Non-functional*	Epithelial integrity: molecular characterization	RNA expression (qPCR, RNA-seq)
		Protein expression (WB, IHC, IF)
	Epithelial integrity: morphological characterization	TEM
		Inflammatory cell death (pyroptosis)

*IFABP, intestinal fatty acid-binding protein; IF, immunofluorescence; IHC, immunohistochemistry; Isc, short-circuit current; LMR, lactulose–mannitol ratio; LPS, Lipopolysaccharide; qPCR, quantitative PCR; TEER, transepithelial electrical resistance; TEM, transmission electron microscopy; WB, Western blot.*

In contrast, the migration of whole commensal bacteria across the intestinal barrier is more likely through transcellular endocytosis in M-cells (Roberts et al., 2010) and enterocytes (Keita et al., 2006; Wu et al., 2014). Lipopolysaccharide (LPS) is located on the surface of Gram-negative bacteria and measured using LPS-binding protein (LBP) as an indication of bacterial translocation. Although markers of epithelial damage such as intestinal fatty acid-binding protein (I-FABP) or locally secreted regulators of intestinal permeability such as zonulin are available (Camilleri and Vella, 2021), the absence of structural defects and methodological limitations of zonulin measurements, respectively, limit their use in FD. Additional *in vivo* approaches include mucosal admittance or impedance measurements using a tissue conductance meter or endoscopic catheter with electrode sensor at the tip (Ishigami et al., 2017; Komori et al., 2019). Alternatively, duodenal manometry combined with impedance allows determination of baseline impedance following the nocturnal migrating motor complex phase III, thus reducing the potential impact of luminal content (Nakagawa et al., 2020). Finally, confocal laser endomicroscopy (CLE) has been used to identify breaks or extrusion zones in the intestinal epithelium through the passage of intravenously administered fluorescein during endoscopy, which are quantified as epithelial gaps or fluorescein leaks (Nojkov et al., 2020). Although increasingly used, the basolateral to apical flux of fluorescein is less physiological than apical to basolateral passage of luminal compounds (Vanuytsel et al., 2021).

### Functional Assessment: *ex vivo*

**FIGURE 2:**
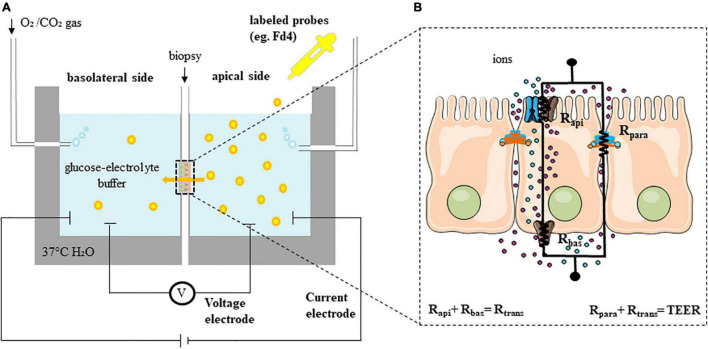
Functional assessment of permeability (*ex vivo*). **(A)** Experimental set-up of Ussing chambers. **(B)** Equivalent electrical circuit model with the transcellular (R_trans_ or sum of apical (R_api_) and basolateral (R_bas_) resistances) and the paracellular (R_para_) resistances in a simple epithelium (TEER is the sum of all individual resistances). Fd4, fluorescein isothiocyanate-labeled 4 kDa dextran; TEER, transepithelial electrical resistance.

In contrast to other tissues, epithelia display two special characteristics: tightness and polarity (Vanuytsel et al., 2021). The presence of TJ separates the apical from the basolateral sides of the enterocyte. Polarity is generated by the asymmetric distribution of channels and transporters to either the apical or the basolateral side of the plasma membrane of the enterocyte. These proteins are responsible among others for the absorption of Na^+^ and secretion of Cl^–^ (Vanuytsel et al., 2021). The gold standard technique for assessing epithelial barrier function is the Ussing chambers, in which the resistance of the epithelium against the passage of ions can be measured by the total transepithelial resistance (R_t_, TEER) (Figure 2A). In general, a monolayer of cells is modeled considering two resistors, R_api_ (resistance of the apical membrane) and R_bas_ (resistance of the basolateral membrane), shunted by a parallel resistor, R_para_ (paracellular resistance exerted by the TJ proteins) (Figure 2B). Then, R_t_ can be calculated by two different approaches using Ohm’s law (ΔV = ΔI × R_t_). Open-circuit conditions are commonly used by injecting a short current pulse (e.g., 16 μA for 200 ms) *via* a resistor and a pair of current electrodes (current clamp) every several seconds or minutes. The device then calculates TEER by measuring the voltage deflection with a high impedance voltmeter and two voltage electrodes. Thus, TEER measures the net flux of all ions across the epithelium with the contribution of para- (R_para_) and transcellular (R_trans_ or the resistance of the apical (R_api_) and basolateral (R_bas_) membranes) resistance. The short-circuit current (Isc) protocol is also used to assess the TEER by applying a constant current to the tissue. Isc is defined as the amount of current per time needed to short-circuit the epithelium and force the potential difference (PD) to be 0 mV. Intermittently, the PD is clamped to values different to 0 mV (voltage clamp) enabling the calculation of the TEER. The Isc reflects the net ion transport movement through the epithelium and can be measured at baseline but also after stimulation with secretagogues such as forskolin (30). Isc measurements are performed when there is an interest of measuring ion transport in the epithelia, mainly the movement of Na^+^ and Cl^–^. Alternatively, the Isc can be also estimated in open-circuit by using Ohm’s law, this is what we know as the equivalent Isc. Most commercially available systems provide both opened-circuit and voltage clamp protocols. The conductance, as the reciprocal of TEER, is also used in the literature (Vanuytsel et al., 2021).

Besides electrophysiology, the flux or passage of labeled probe molecules such as dextrans, EDTA, mannitol, inulin and PEG can be determined after application at the luminal side and serial sampling from the basolateral side (Vanuytsel et al., 2021). Because of their size, most of these molecules reflect the paracellular leak pathway, where molecules with a radius of 20 Å or size ranging from 4 to 20 kDa can permeate in the intestinal crypts, regardless of charge (Camilleri, 2019; Shen et al., 2011). In contrast, the size- and charge-selective pore pathway enables high-capacity transport of ions and solutes with a radius up to 4 Å and is predominant near the tips of the villi (Shen et al., 2011; Zuo et al., 2020). Whereas fluorescein isothiocyanate [FITC]-dextran (Fd4, 4 kDa) is the most commonly used molecule to assess the paracellular leak pathway, larger molecules (e.g., horseradish peroxidase (HRP, 44 kDa) or dextran-labeled molecules ranging from 40–80 kDa) can be used to assess the transcellular passage (Table 1; Vanuytsel et al., 2021). As mentioned above, bacterial passage occurs through transcellular endocytosis, which can be measured in duodenal biopsies using chemically killed and fluorescein-conjugated *Escherichia coli* (*E. coli*) or other bacteria, of which antigenicity is retained (Beeckmans et al., 2020). Alternative bacteria such as *Salmonella typhimurium* have been studied using colonic biopsies in irritable bowel syndrome (IBS) patients (Bednarska et al., 2017). Following fixation of the exposed biopsies, bacterial passage can be confirmed using fluorescence and transmission electron microscopy (TEM; Bednarska et al., 2017; Beeckmans et al., 2020).

### Non-functional Assessments

Functional alterations of the epithelial barrier function assessed *in vivo* and *ex vivo* can be further characterized at molecular level by studying the expression of cell-to-cell adhesion proteins at mRNA and protein levels. We have to be cautious to state, based only on the expression of these genes (molecular biology data), that the epithelial barrier is functionally compromised. In other words, up and downregulation of some cell-to-cell adhesion proteins are not always associated with functional alterations (Weber et al., 2010). Importantly, only TJ proteins directly regulate the paracellular permeability to ions and larger molecules. An altered expression of AJ and desmosomal proteins can influence the expression of TJ proteins but do not directly regulate the paracellular transport. Several studies have used quantitative PCR (qPCR) for TJ (OCLN, CLDN, ZO), AJ (E-cadherin, β-catenin) and desmosomal proteins (DSG, DSC) (Vanheel et al., 2014; Komori et al., 2019; Taki et al., 2019; Nojkov et al., 2020). Also, analysis of protein-expression of cell-to-cell adhesion molecules by western blot (WB) and semi-quantitative evaluation of immunohistochemically (IHC) stained duodenal biopsies have been done in FD (Vanheel et al., 2014; Du et al., 2018). With the development of next-generation sequencing techniques including RNA-seq, a transcriptomic analysis of whole tissues, including duodenal biopsies, has recently been performed (Puthanmadhom Narayanan et al., 2021b). This included analysis of micro-RNA (miRNA), which may regulate epithelial barrier genes (Puthanmadhom Narayanan et al., 2021a,b).

A structural analysis of the apical junction complex in microvilli is possible but very time consuming using TEM, with assessment of the distance between two adjacent enterocytes, proportions of dilated junctions (>20 nm) and junctions with perijunctional cytoskeleton condensation which blur the intercellular membranes (Puthanmadhom Narayanan et al., 2021b). These measures are, however, not standardized, with different definitions of an altered distance between adjacent enterocytes (up to 30 nm) or lack of separate assessment at the TJ, AJ and desmosomal complexes (Tanaka et al., 2016). Finally, markers of inflammatory cell death or pyroptosis (caspase-1) have been used with IHC to detect potential extrusions of duodenal epithelial cells in response to environmental stimuli (Nojkov et al., 2020).

## Duodenal Hyperpermeability in Functional Dyspepsia

### Functional Assessment: *in vivo*

Studies using differential urinary sugar excretion tests in FD are scarce (Table 2). In pediatric FD patients, a sugar absorption test using lactulose, mannitol and sucrose with a 5 h-urine collection was similar to controls, although the study was only powered to detect a 3-fold difference in LMR (Neilan et al., 2014). In adult FD patients, LMR between 1 and 2 h was greater vs. controls, even after adjusting for anxiety and depression with no difference between PDS and EPS (Puthanmadhom Narayanan et al., 2021b). While the choice for 1–2 h excretion was based on the delayed gastric emptying of liquids in FD, it should be noted that gastric emptying is measured with solids whereas the permeability probe is administered in fasted state and this modification of the technique is not validated (Wauters et al., 2021a). Indeed, previous reports on the same cohort reported no difference in 0–2 h LMR (Desai et al., 2019). Although LMR is not specific for duodenal permeability, a correlation was found with Fd4-passage (see “Functional Assessment: *ex vivo*”) (Puthanmadhom Narayanan et al., 2021b). Of note, urinary sucrose excretion was similar in dyspeptic Japanese patients (based on Rome III criteria without endoscopy) vs. controls after a 4 h-urine collection (Nakae et al., 2016). It should, however, be noted that sucrose excretion was previously shown to be affected by different drugs (Mujagic et al., 2014).

**TABLE 2:** Impaired duodenal mucosal permeability in adult Functional Dyspepsia patients.

Findings		Methods	Population	Trial details
***In vivo* permeability**				
↑LMR (60–120 min)	∼↑Fd4	HPLC-MS	39 NUD (16 on-PPI) vs. 24 controls	United States, 2021 (Puthanmadhom Narayanan et al., 2021b)
↑Mucosal admittance		tissue conductance	21 FD (Rome III, 17 on-PPI) vs. 23 controls	Japan, 2017 (Ishigami et al., 2017)
↓Mucosal impedance	∼↓ZO1, IL-1β	tissue conductance	24 FD (Rome III, 12 on-PPI) vs. 20 controls (1 on-PPI)	Japan, 2019 (Komori et al., 2019)
↓Baseline impedance		HRM/Z	16 FD (Rome IV, 1 Hp-positive) vs. 15 controls	United Kingdom, 2020 (Nakagawa et al., 2020)
↑Epithelial gap density (D3)		CLE	14 FD (Rome IV, 3 on-PPI) vs. 8 controls	United States, 2020 (Nojkov et al., 2020)
***Ex vivo* permeability**				
↑Isc (resting and stimulated)		Ussing chambers	37 NUD (30 Rome III, 15 on-PPI) vs. 20 controls	United States, 2021 (Puthanmadhom Narayanan et al., 2021a)
↑Fd4-passage, ↓TEER		Ussing chambers	15 FD (Rome III, 6 on-PPI) vs. 15 controls	Belgium, 2014 (Vanheel et al., 2014)
↑Fd4-passage, ↓bacterial passage		Ussing chambers	FD	Belgium, 2020 (Beeckmans et al., 2020)
↓TEER	∼ Abdominal pain, bloating, IFNγ	Ussing chambers	10 FD (Rome IV, 3 on-PPI) vs. 10 controls (globus/IDA, 4 on-PPI)	United States, 2020 (Nojkov et al., 2020)
↑Fd4-passage		Ussing chambers	28 FD (Rome IV) vs. 30 controls	Belgium, 2021 (Wauters et al., 2021b)
**Non-functional assessment**				
↓ZO1 (protein) ↓OCLN (RNA, protein), p-OCLN (protein) ↓β-catenin (RNA, protein), E-cadherin (protein) ↓DSC2 (RNA), DSG2 (RNA, protein)	p-OCLN ∼ ↑eosinophils, ↑mast cells, ↓TEER E-cadherin ∼ ↑mast cells, ↑eosinophils, ↑Fd4	qPCR, WB, IF	15 FD (Rome III, 6 on-PPI) vs. 15 controls	Belgium, 2014 (Vanheel et al., 2014)
↓ZO1 (RNA)	∼↓Impedance	qPCR	24 FD (Rome III) vs. 20 controls	Japan, 2019 (Komori et al., 2019)
↓CLDN1 (RNA)		qPCR	10 FD (Rome IV, 3 on-PPI) vs. 10 controls (globus/IDA, 4 on-PPI)	United States, 2020 (Nojkov et al., 2020)
↓CLDN1 (protein)		IHC	9 FD (Rome III) vs. 9 controls	China, 2018 (Du et al., 2018)
↑CLDN3 (RNA)		qPCR	35 FD (Rome III, 7 Hp-positive) vs. 31 controls (3 Hp-positive)	Japan, 2019 (Taki et al., 2019)
↓ZO1, OCLN, CLDN12, CLDN18 ↓E-cadherin, p120 catenin, nectin-3 ↓DSG2, DSC2, plakophilin-2, plakoglobin ↓/↑ regulatory miRNAs		RNA-seq	39 NUD (32 Rome III, 16 on-PPI) vs. 21 controls	United States, 2021 (Puthanmadhom Narayanan et al., 2021b)
↓Junctions with perijunctional condensation	Intercellular distance and intercellular distance ∼Fd4-passage, ZO2/3 junctions with perijunctional condensation ∼ZO2	TEM	37 NUD (32 Rome III, 16 on-PPI) vs. 21 controls	United States, 2021 (Puthanmadhom Narayanan et al., 2021b)
↑Intercellular paracellular distance (adherens junction)	∼Postprandial fullness, early satiety	TEM	9 FD (Rome III) vs. 5 controls (1 Hp-positive)	Japan, 2016 (Tanaka et al., 2016)
↑Pyroptosis (caspase-1)		IHC	14 FD (Rome IV, 3 on-PPI) vs. 6 controls (globus/IDA, 4 on-PPI)	United States, 2020 (Nojkov et al., 2020)

*CLDN, claudin; CLE, confocal laser endomicroscopy; DIS, dilated intercellular spaces; DSC, desmocollin; DSG, desmoglein; Fd4, fluorescein isothiocyanate-labeled 4 kDa dextran; Hp, Helicobacter pylori; HPLC-MS, high-performance liquid chromatography-mass spectrometry; HRM/Z, high-resolution manometry impedance; IDA, iron deficiency anemia; IF, immuno-fluorescence; IFN, interferon; IHC, immuno-histochemistry; IL, interleukin; Isc, short-circuit current; NUD, non-ulcer dyspepsia; OCLN, occludin; PPI, proton pump inhibitor; qPCR, quantitative PCR; TEER, transepithelial electrical resistance; TEM, transmission electron microscopy; WB, Western blot; ZO, zonula-occludens. ∼ for correlation, underlined text for significant changes after correction for multiple testing.*

**FIGURE 3:**
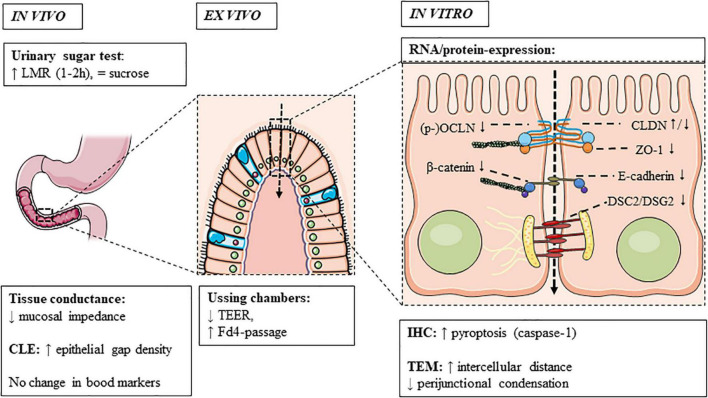
Duodenal permeability in functional dyspepsia. Increased permeability is observed in functional dyspepsia using *in vivo* and *ex vivo* techniques and associated with a dysregulation of cell-to-cell adhesion proteins, of which the main findings are summarized. CLDN, claudin; CLE, confocal laser endomicroscopy; Fd4, fluorescein isothiocyanate-labeled 4 kDa dextran; IHC, immunohistochemistry; OCLN, occludin; TEER, transepithelial electrical resistance; TEM, transmission electron microscopy; ZO, zonula-occludens.

In adult FD, duodenal mucosal impedance was decreased vs. controls (Ishigami et al., 2017; Komori et al., 2019; Nakagawa et al., 2020). While the consistently lower but gradual increase in baseline impedance from the duodenum to jejunum in FD patients and controls was probably due to a progressive decrease in exposure to luminal content (Nakagawa et al., 2020), a higher epithelial gap density was only present in the distal duodenum of FD vs. controls using CLE (Nojkov et al., 2020). Although these cell extrusions were suggested to allow microbial passage in the duodenum, we found no differences in plasma LBP as a marker of intestinal bacterial translocation in FD (Wauters et al., 2021b). Moreover, challenges remain in the interpretation and reproducibility of these novel methods, as no correlation was found between CLE and *ex vivo* permeability (Nojkov et al., 2020). In contrast, mucosal impedance correlated with *ex vivo* permeability (Ishigami et al., 2017), as well as decreased ZO1 and increased IL-1β expression (see “Non-functional Assessments”) (Komori et al., 2019). Zonulin was not increased in functional GI patients, which may be related to technical limitations of the assay (Talley et al., 2020). In conclusion, current *in vivo* measurements do not fully assess duodenal permeability in FD, as demonstrated *ex vivo* and *in vitro* and as discussed in the next sections (Figure 3).

### Functional Assessment: *ex vivo*

The first study using Ussing chambers showed a lower TEER and higher Fd4-passage in adult FD patients vs. controls (Vanheel et al., 2014). Recently, increased Fd4-passage was confirmed in an independent cohort by our group, with no changes in TEER, which was similar to studies from the United States (Puthanmadhom Narayanan et al., 2021a,b). Indeed, altered epithelial secretion in FD may influence TEER (Puthanmadhom Narayanan et al., 2021a), as the applied current for measuring the generated potential and thus resistance is carried by the common ions Na^+^ and Cl^–^ (Shen et al., 2011). Although decreased TEER was reported by others, the methodology differed and Fd4-passage was not measured (Nojkov et al., 2020). Compared to the lack of association between TEER and *in vivo* permeability (Nojkov et al., 2020), the importance of Fd4-passage is illustrated by the correlations with *in vivo* (LMR) and *in vitro* (intercellular distance and dilated junctions) permeability in FD patients (Puthanmadhom Narayanan et al., 2021b), with the majority of molecular changes pointing to the paracellular leak pathway (see “Non-functional Assessments”). This would indeed suggest an increased paracellular influx of relatively larger molecules into the duodenal mucosa, potentially resulting in immune activation as discussed below.

Increased *ex vivo* permeability and correlations with (the ratio of) primary and secondary bile salts in FD were found but did not persist after correction for multiple testing (Beeckmans et al., 2020). The lack of correlations between duodenal permeability and (conjugated) bile salts was later confirmed and not unexpected as direct but non-physiological epithelial effects are only expected for (unconjugated) bile acids (Wauters et al., 2021f). Indeed, potential cytotoxic effects of hydrophobic bile acids were found in human colonic biopsies and the murine small intestine (Münch et al., 2007; Forsgård et al., 2014), with increased bacterial uptake in patients with collagenous colitis in remission (Münch et al., 2011). In addition to paracellular permeability, increased transcellular passage of bacteria was described in the colonic epithelium of IBS (Bednarska et al., 2017). While this may contribute to recurrence of colonic inflammation in IBS (Münch et al., 2011), similar mechanisms have not yet been shown in the duodenum of FD patients with even lower transcellular duodenal passage of *E. coli* in FD vs. controls (Beeckmans et al., 2020).

### Non-functional Assessments

The expression of cell-to-cell adhesion proteins was decreased at the gene and protein level in adult FD patients (Table 2). We previously showed decreased gene and/or protein expression of TJ (ZO1 and OCLN) or AJ (β-catenin and E-cadherin) and desmosomal proteins in FD patients vs. controls (Vanheel et al., 2014). Recently, lower gene-expression of TJ (ZO1, OCLN, CLDN12, and CLDN18) or AJ (E-cadherin, p120 catenin and nectin-3) and desmosomal proteins (DSG2, DSC2, plakophilin-2 and plakoglobin) were confirmed in dyspeptic patients using RNA-seq (Puthanmadhom Narayanan et al., 2021b). In addition, differential expression of 11 miRNAs that regulate epithelial barrier genes was found (Puthanmadhom Narayanan et al., 2021b). Of these, MiR-144, the human equivalent of which was up-regulated in FD patients (Puthanmadhom Narayanan et al., 2021a,b), inhibited the expression of ZO1 and OCLN in a rat model of IBS (Hou et al., 2017). As the decreased gene expression of ZO1 was correlated with duodenal mucosal impedance and Fd4-passage in FD patients (Desai et al., 2019; Komori et al., 2019), these changes suggest involvement of the paracellular leak pathway (Figure 3). In contrast, CLDN transmembrane proteins were unaltered at the gene and protein level (Vanheel et al., 2014; Komori et al., 2019; Taki et al., 2019; Nojkov et al., 2020), except for decreased CLDN1 (Du et al., 2018; Nojkov et al., 2020) and increased CLDN3-expression (Taki et al., 2019). Therefore, involvement of the pore pathway is less likely in the pathophysiology of FD.

Besides molecular changes, structural alterations were found with a lower proportion of junctions with perijunctional cytoskeleton condensation (Puthanmadhom Narayanan et al., 2021b). Moreover, the intercellular distance and proportion of dilated junctions correlated with Fd4-passage and the expression of TJ proteins (Puthanmadhom Narayanan et al., 2021b), also pointing to involvement of the leak pathway (Figure 3). Another study reported dilated junctions at the AJ and not TJ or desmosomes, which correlated with PDS-like symptoms (Tanaka et al., 2016). Interestingly, similar alterations were observed in the jejunum of diarrhea-predominant IBS patients, which correlated with bowel habits and symptoms (Martínez et al., 2013). Increased colonic permeability in IBS was associated with mast cell activation (see “Link With Mucosal Inflammation”) (Martínez et al., 2012; Martínez et al., 2013). In addition, esophageal spongiosis or dilated intercellular spaces, as well as increased *in vivo* and *ex vivo* permeability in eosinophilic esophagitis (EoE) correlated with mucosal eosinophils (Warners et al., 2017). As increased small intestinal permeability in active EoE was found by some (Katzka et al., 2015) but not others (Warners et al., 2017), similar interactions with eosinophils may occur in FD as discussed below. Recently, increased inflammatory epithelial cell death or pyroptosis was described, which could account for epithelial gaps measured with CLE (Nojkov et al., 2020). However, associations with other *in vivo* and *ex vivo* or *in vitro* measurements and validation is lacking.

## Factors Related to Intestinal Permeability

### Link With Mucosal Inflammation

We have recently reviewed the role of the duodenum in FD, in which we hypothesized that loss of mucosal integrity may lead to immune activation through antigen presentation with a T-helper type 2 (Th2) response, leading to the infiltration of eosinophils and mast cells (Wauters et al., 2020b). Indeed, we previously found correlations between the reduced protein expression of p-OCLN and E-cadherin with increased eosinophils and mast cells in FD (Vanheel et al., 2014). Although the number and degranulation of duodenal eosinophils were not correlated with *ex vivo* permeability in a follow-up study (Vanheel et al., 2018), under-detection of degranulation is possible using MBP-based methods (Jiménez-Saiz et al., 2020). We also described the Biobreeding (BB)-rat as a spontaneous animal model of DGBI, in which an eosinophil-predominant mucosal inflammation was preceded by increased permeability in the jejunum (Vanuytsel et al., 2014a). Interestingly, the release of MBP from eosinophils decreased the expression of OCLN with decreased colonic barrier function in culture and animal models (Furuta et al., 2005). Similarly, eosinophils have been linked to colonic barrier dysfunction in IBD, which was also present in remission and might exacerbate colonic inflammation (Wallon et al., 2011; Katinios et al., 2020). Therefore, reciprocal interactions exist between eosinophil infiltration and increased permeability in DGBI, IBD as well as EoE as discussed above, suggesting similar mechanisms in the duodenum of FD patients.

While colonic mast cells are commonly linked to IBS and IBD (Wallon et al., 2011; Wouters et al., 2016), higher numbers with a similar degree of degranulation were also found in the duodenum of FD patients vs. controls (Vanheel et al., 2014; Vanheel et al., 2018), pointing to a potential role in FD. Despite a more heterogeneous cellular profile, no association was found with duodenal permeability (Vanheel et al., 2018). Mast cell-derived tryptase cleaved and activated protease-activated receptor (PAR) 2 on colonocytes, with decreased expression of OCLN and ZO1 and increased *ex vivo* permeability (Jacob et al., 2005). In contrast, no differences in duodenal PAR-expression or correlations with *in vivo* permeability were found in FD patients (Komori et al., 2019). Nevertheless, tryptase and PAR2 may mediate eosinophil migration (Matos et al., 2013) and play a role in FD pathogenesis, as tryptase was also upregulated after duodenal acid infusion in healthy individuals resulting in increased *ex vivo* permeability (Vanheel et al., 2020). Moreover, mast cells were involved in stress-induced small intestinal hyperpermeability (see “Local and Systemic Interactions”) (Vanuytsel et al., 2014b), which was associated with decreased IFNγ and increased IL4 expression or Th2-type reactions with eosinophil infiltration in animal models (Yang et al., 2006). In the absence of specific treatments targeting permeability or inflammation, clinical research is, however, limited to associations between duodenal mucosal barrier and immune dysfunction in FD. To the best of our knowledge, the BB-rat is the only available animal model which demonstrates gastric dysmotility, small intestinal hyperpermeability and eosinophilia (Vanuytsel et al., 2014a). Therefore, mechanistic evidence from preclinical studies with similar alterations to FD patients is lacking as recently reviewed (Accarie and Vanuytsel, 2020).

### Local and Systemic Interactions

While no causality or directionality has yet been established between permeability and inflammation, the link with symptoms is likely to be *via* neuronal changes, as functional and structural neuronal alterations have been described in FD (Cirillo et al., 2015). Besides a correlation between neuronal changes and mucosal eosinophils or mast cells (Cirillo et al., 2015), upregulated glial cell line-derived neurotrophic factor (GDNF) may also play a role in the protection of a disturbed epithelial barrier in FD (Tanaka et al., 2016). In IBS, reactive enteric glial cells have been linked to bacterial translocation in the colon *via* mast cells with different profiles in health (MeiradeFaria et al., 2021), suggesting a role for neuro-immune interactions. While no link was found between *ex vivo* permeability and symptoms (Vanheel et al., 2014; Wauters et al., 2021b), the central interactions are complex as illustrated by an inverse association with increased functional and structural connectivity in endogenous pain facilitation regions in IBS patients (Witt et al., 2019). Although similar studies are lacking in FD, increased duodenal permeability (Fd4 passage) was linked with gastric emptying in FD patients (Wauters et al., 2021f), supporting the concept of altered duodenogastric feedback in dyspeptic symptom generation (Wauters et al., 2020b). Moreover, increased gut-homing lymphocytes have been linked with gastric emptying and symptoms in FD (Liebregts et al., 2011). However, duodenal barrier dysfunction has not yet been linked to systemic inflammation and despite increased high-sensitivity CRP, no increase in LBP or duodenal bacterial passage was found in FD (Beeckmans et al., 2020; Wauters et al., 2021b).

Besides a possible role for luminal factors in FD, including duodenal acid and bile (Wauters et al., 2021f), the loss of mucosal integrity may also be triggered by stress-induced activation of immune cells (Zheng et al., 2009). During stress, the hypothalamus is not the only source of corticotropin-releasing hormone (CRH). Intestinal eosinophils produce and release CRH, which activates mast cells and in turn increases intestinal permeability (Zheng et al., 2009). Small intestinal permeability is also increased by intravenous administration of CRH and blocked by pre-treatment with a mast cell stabilizer in healthy students, even after stress (public speech) (Vanuytsel et al., 2014b). CRH was released by mucosal eosinophils and activated mast cells in the colon (Wallon et al., 2008; Wallon et al., 2011), and both were implicated in stress-mediated dysfunction of the jejunum in IBS (Guilarte et al., 2020; Salvo-Romero et al., 2020). Activation of mucosal immune cells with increased permeability is also possible *via* food or microbial triggers with a role for local IgE-antibodies as a mechanism for food-induced abdominal pain (Aguilera-Lizarraga et al., 2021). Although the potential role of food allergens in FD is still unclear, duodenal mucosal nutrient challenge followed by CLE showed positive reactions in 70% of IBS patients, of which 61% reacted to wheat (Fritscher-Ravens et al., 2019). Changes in permeability include increased CLDN2- and decreased OCLN-expression, as well as an increased intra-epithelial lymphocytes (IEL) and eosinophil degranulation, suggestive of an atypical or non-IgE mediated food allergy (Fritscher-Ravens et al., 2019). Although these mechanisms have not yet been studied in FD, the decreased duodenal p-OCLN-expression was previously correlated with duodenal eosinophils (Vanheel et al., 2014). These findings also point to the involvement of the leak pathway.

## Effect of Existing and Novel Treatments

Besides acid-suppressive effects of proton pump inhibitors (PPI) as current first-line therapy for FD, we recently described the first prospective evidence for PPI-induced reductions in permeability and inflammation (eosinophils and mast cells) in FD patients (Wauters et al., 2021b). Although the increased duodenal Fd4-passage was normalized after PPI-therapy, only anti-eosinophil and not barrier-protective effects were associated with clinical efficacy of PPI (Wauters et al., 2021b). Besides increased duodenal mucosal permeability and inflammation in FD patients vs. controls off-PPI, similar alterations were found in FD patients with refractory symptoms after >1 month on-PPI (Wauters et al., 2021b). Importantly, no baseline differences in permeability were found between FD patients off- (PPI-naïve) or on-PPI (refractory), illustrating the limitation of cross-sectional studies, which do not account for short- vs. long-term use of PPI (Wauters et al., 2021b). Indeed, previous studies did not assess potential PPI-effects on *ex vivo* and *in vivo* permeability prospectively (Vanheel et al., 2014; Komori et al., 2019; Nojkov et al., 2020). We also showed a reduction in salivary cortisol as a marker of stress in FD patients after PPI, with no association between changes in cortisol and Fd4-passage (Wauters et al., 2021b). Although a preclinical study reported that PPI enhanced the stress-induced increase in small intestinal permeability *via* dysbiosis (Takashima et al., 2020), these findings cannot be translated to patients (Ceulemans et al., 2020). As PPI may influence different factors, including the duodenal microbiome (Wauters et al., 2021e), future studies with more targeted therapies are needed.

Interestingly, a low fiber (<10 g/day) and high simple sugar (>50% of daily carbohydrates) diet triggered functional GI-symptoms with increased duodenal Fd4-passage and decreased microbial diversity in healthy subjects (Saffouri et al., 2019). Moreover, both symptoms and duodenal permeability were linked with decreased bacterial diversity (Saffouri et al., 2019). In addition, a randomized placebo-controlled trial of glutamine supplements showed that the reduction in intestinal hyper-permeability correlated with improved clinical outcomes in diarrhea-predominant IBS patients (Zhou et al., 2019). Although similar effects have not yet been studied in FD, barrier-protective effects of probiotics for NSAID-induced enteropathy were mainly found in the duodenum, suggesting potential benefits (Mortensen et al., 2019). While beneficial effects of *L. plantarum* strains were found on duodenal mucosal gene transcription, LMR was unaffected after NSAID-administration in healthy volunteers (Mujagic et al., 2017). While no effects were found of a *Lactobacillus gasseri* strain (LG21) on urinary sucrose excretion in dyspeptic patients (Nakae et al., 2016), the probiotic *L. casei* LC01 regulated epithelial permeability through reduced miR-144 and increased OCLN and ZO1 expression (Hou et al., 2020). Interestingly, beneficial *in vitro* effects of gastric acid-resistant endospores on permeability and inflammation may also contribute to the immunological and microbial benefits of *Bacillus coagulans* and *subtilis* strains in FD (Marzorati et al., 2020; Wauters et al., 2021d). Similar to our previous study, no effects on LBP were found with these spore-forming probiotics (Wauters et al., 2021d), even in combination with other pro- and prebiotics in long-term PPI-users (Horvath et al., 2020). Indeed, alterations in FD patients on long-term PPI would justify the search for alternative treatments, including pre- or probiotics.

## Conclusion

Functional dyspepsia is a common disorder with unknown pathophysiology, hampering a conclusive diagnosis and the development of effective drugs. Multiple independent findings using different methods provided evidence for duodenal barrier dysfunction in FD patients. Involvement of the paracellular leak pathway measured in Ussing chambers (Fd4-passage) is further supported by correlations with *in vivo* (LMR) permeability, with decreased expression of TJ-related proteins. As these molecular changes are correlated with increased immune cells, antigen penetration *via* a defective barrier could indeed result in mucosal inflammation. Although associations do not prove causality, both increased permeability and inflammation most likely amplify each other, leading to a vicious circle. In the absence of specific barrier-protective drugs, future studies should include assessment of duodenal permeability in relation to symptoms and treatment of FD.

## Author Contributions

LW wrote the original draft. All authors contributed to the manuscript, revised and approved the final version of the review.

## Conflict of Interest

LW reports financial support for research from Danone and MyHealth; has served on the advisory board of Naturex; has served on the Speaker bureau for Dr. Falk Pharma, Takeda and MyHealth. TV reports financial support for research from Danone, MyHealth, Takeda and VectivBio; has served on the Speaker bureau for Abbott, Dr. Falk Pharma, Fresenius Kabi, Menarini, Remedus, Takeda, Truvion, and VectivBio; reports consultancy fees from Baxter, Dr. Falk Pharma, Takeda, VectivBio, and Zealand Pharma. JT has given Scientific advice to Adare, AlfaWassermann, Arena, Bayer, Christian Hansen, Clasado, Danone, Devintec, Falk, FitForMe, Grünenthal, Ironwood, Janssen, Kiowa Kirin, Menarini, Mylan, Neurogastrx, Neutec, Novartis, Nutricia, Reckitt Benckiser, Ricordati, Shionogi, Takeda, Truvion, Tsumura, Zealand, and Zeria pharmaceuticals, has received research support from Biohit, Shire, Sofar, and Takeda, and has served on the Speaker bureau for Abbott, Allergan, AstraZeneca, FitForMe, Janssen, Kyowa Kirin, Mayoly, Menarini, Mylan, Novartis, Schwabe Parmaceuticals, Takeda, Wellspect, and Zeria. All the fundings reported were outside of this study. The remaining authors declare that the research was conducted in the absence of any commercial or financial relationships that could be construed as a potential conflict of interest.

## Publisher’s Note

All claims expressed in this article are solely those of the authors and do not necessarily represent those of their affiliated organizations, or those of the publisher, the editors and the reviewers. Any product that may be evaluated in this article, or claim that may be made by its manufacturer, is not guaranteed or endorsed by the publisher.

## Abbreviations

AJ: adherens junctions
CLDN: claudin
CLE: confocal laser endomicroscopy
CRH: corticotrophin-releasing hormone
DGBI: disorders of gut-brain interaction
DSC: desmocollin
DSG: desmoglein
EoE: eosinophilic esophagitis
EPS: epigastric pain syndrome
Fd4: fluorescein isothiocyanate-labeled 4 kDa dextran
FD: functional dyspepsia
GI: gastrointestinal
IBD: Inflammatory bowel disease
IBS: Irritable bowel syndrome
IHC: immunohistochemistry
Isc: short-circuit current
LBP: Lipopolysaccharide-binding protein
LMR: lactulose–mannitol ratio
LPS: Lipopolysaccharide
MUC2: Mucin 2
OCLN: occludin
PD: potential difference
PDS: Postprandial distress syndrome
PPI: Proton pump inhibitor
qPCR: quantitative PCR
STAT: Signal transducer and activator of transcription
TEER: transepithelial resistance
TEM: transmission electron microscopy
TJ: Tight junction
ZO: zonula occludens.
